# Epidemiological characteristics of human rabies in Henan province in China from 2005 to 2013

**DOI:** 10.1186/s40409-015-0034-7

**Published:** 2015-09-02

**Authors:** Guo Wei Li, Qiao Ge Chen, Zhen Yu Qu, Yao Xia, Alfred Lam, Ding Mei Zhang, Jia Hai Lu

**Affiliations:** Department of Medical Statistic and Epidemiology, School of Public Health Zhongshan School of Medicine, Sun Yat-sen University, 74 Zhongshan Road II, Guangzhou, 510080 Guangdong China; Department of Immunology, Zhengzhou Center for Disease Control and Prevention, Zhengzhou, Henan China; Department of Anatomy, Henan Medical College, Zhengzhou, Henan China; Department of Pathogenic Biology and Immunology, Luohe Medical College, Luohe, Henan China; Department of Pathology, Menzies Health Institute Queensland, Griffith University, Gold Coast, QLD Australia

**Keywords:** Human rabies, Zoonosis, Epidemiology, Dog bite

## Abstract

**Background:**

Rabies is very common in People’s Republic of China. Henan province, in the central portion of China, is one of the most densely populated provinces in the entire country. With the new rabies epidemic trend noted in northern and western China, it is necessary to investigate the characteristics of human rabies in this area and control the disease.

**Methods:**

We chose patients in hospital isolation in 18 municipalities in Henan province as the investigation subjects. Data were collected through systematic reporting from these hospitals, whereas a questionnaire was applied to the relatives of patients.

**Results:**

A total of 1022 rabies cases were reported. The incidence of human rabies in Henan has increased rapidly since 2005, having peaked in 2007, and maintained a high level in the remaining years. The cases were found mainly in rural areas in the south and east of the province. Rabies was often noted in summer and with the highest number in August. Most cases were noted in males and often in farmers. The patients aged between 40 and 60 years accounted for 36.8 % of all the cases. The wound treatment rate (12.2 %) and vaccination rate (2.6 %) of rabies cases after exposure were relatively low, while the rabies immunoglobulin utilization rate was only 2.8 %.

**Conclusions:**

Rabies epidemic cases at the county level overall were increasing in Henan province during the period of 2005–2013; the epidemic has spread quickly. The data in this study imply that the disease could be better managed by more integrated surveillance across human health and veterinary sectors, improved education and better government policies.

## Introduction

Rabies is a zoonosis with a mortality rate in humans approaching 100 %. The disease is very common in the People’s Republic of China, which has noted more cases of human rabies than any other country except India. Henan, in the central portion of China, is one of the most densely populated provinces in China. Given the recent trend of Chinese rabies epidemic noted in northern and western China, the risk of a nationwide epidemic is greater [1, 2]. In 2000, there was only one case of rabies in Henan. The case number increased to 46 in 2003 and was as high as 130 in 2004 [3, 4]. To investigate the epidemic situation of rabies in Henan province, China, and to explore a strategy for prevention and control of rabies, we collected and analyzed rabies epidemic data and the information on the treatment of the human rabies case in the province from 2005–2013.

## Methods

### Data sources

We choose patients who were clinically diagnosed with rabies according to the “diagnostic criteria for rabies” (ratified by Ministry of Health) [5]. In isolation hospitals (sentinel hospitals) of 18 municipalities, from 2005–2013, data of investigation subjects were collected through systematic reporting whereas questionnaires were administered to the relatives of patients.

### Statistical treatment

The data were entered into an Excel file (version 2003, Microsoft Corporation, USA). MapInfo Professional (version 10, Pitney Bowes Ltd, USA) was used for spatial analysis. Statistical analysis was performed using the Statistical Package for Social Sciences for Windows (version 22.0, IBM SPSS Inc., USA).

## Results

### The human rabies incidence

Henan province is situated in the mid-eastern region of China between northern latitudes 31°23–36°22 and eastern longitudes of 110°21–116°39. The climate spans from warm temperate to subtropical, humid to semi-humid with risk for monsoons, and has average annual temperatures spanning 12 °C-16 °C. The province occupies an area of 165,994 km^2^ divided into 18 municipalities, which are subdivided into 159 county-level divisions. Its population was reported to be 94 million in 2010 [6]. The population numbers in the 18 municipalities from 2005–2013 are shown in Table 1.

**Table 1:** The number of population (*ten thousand*) in 18 municipalities from 2005 to 2013

Municipality	2005	2006	2007	2008	2009	2010	2011	2012	2013
Zhengzhou	68	68	68	67	68	68	86	87	89
Kaifeng	46	47	47	47	47	48	47	47	48
Luoyang	64	63	64	63	64	65	65	66	65
Pingdingshan	49	49	49	49	49	50	49	49	51
Anyang	53	54	53	53	53	54	51	53	54
Hebi	14	14	14	14	14	14	14	14	14
Xinxiang	55	56	55	55	56	56	57	57	56
Jiaozuo	33	33	33	33	33	34	35	35	35
Puyang	36	36	36	35	36	36	36	37	36
Xuchang	42	42	42	41	42	42	43	43	42
Luohe	23	23	23	22	23	23	25	25	25
Sanmenxia	22	22	22	22	22	22	22	22	22
Nanyang	98	99	99	98	99	100	102	100	101
Shangqiu	79	80	79	79	80	81	73	75	73
Xinyang	66	66	66	65	66	66	61	62	61
Zhoukou	100	100	100	100	101	102	89	90	92
Zhumadian	76	76	76	76	76	77	72	71	70
Jiyuan	6	7	6	7	6	7	6	7	7
Total	930	935	932	926	935	945	933	940	941

**Fig. 1:**
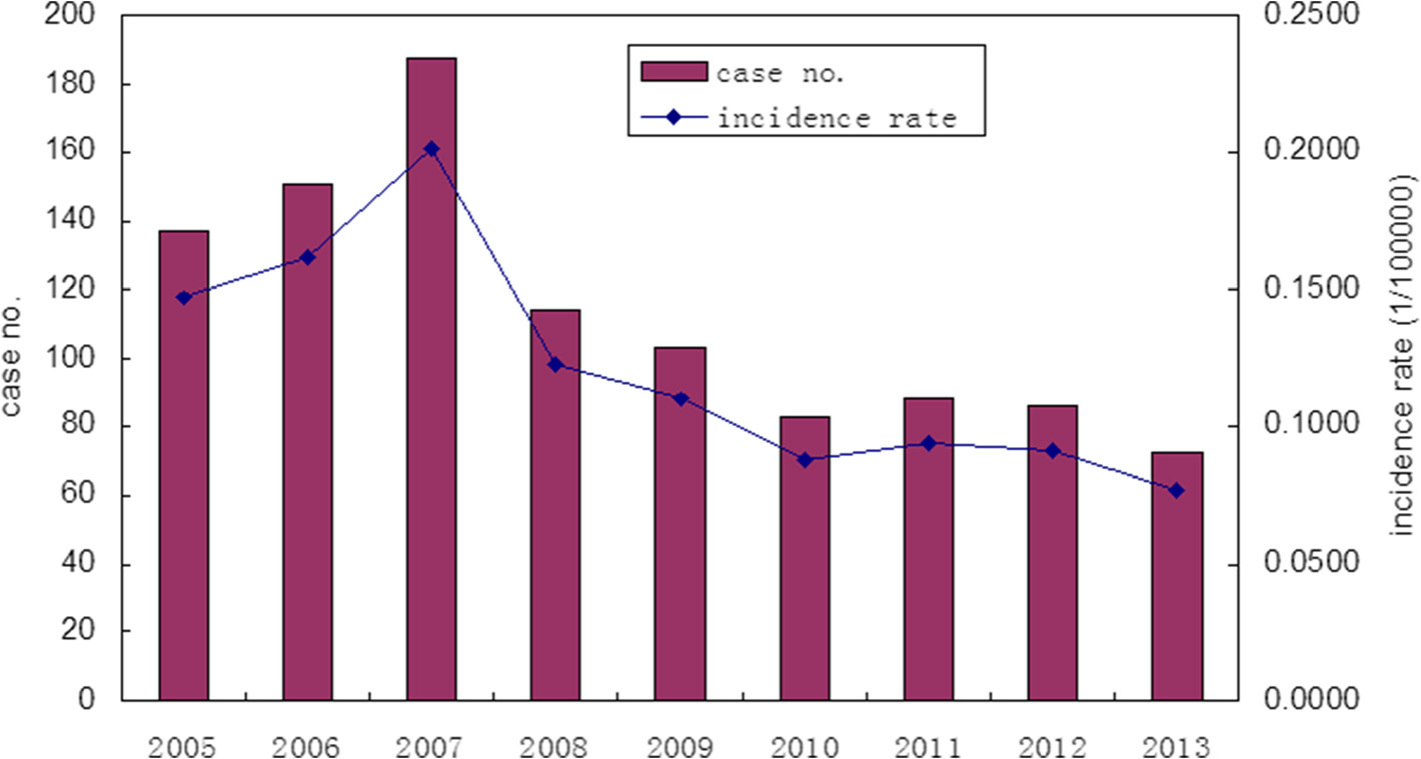
The rabies incidence trends in Henan province from 2005 to 2013

Henan had recorded a total of 1022 rabies cases from 2005–2013; the average annual incidence of the disease in this period was 0.12 per 100,000 inhabitants. There was an upward trend from 2005–2007, reaching a peak in 2007. The numbers of counties (districts) reported cases increased from 52 in 2005–89 in 2007. The epidemic spread rapidly in the whole province. After the 2007 outbreak, the number of cases declined. The decline accelerated from 2007–2010. Then, there was a slight increase from 2010–2012 but the number of cases of rabies reached the lowest value in 2013 (Fig. 1).

### Regional distribution of human rabies

**Fig. 2:**
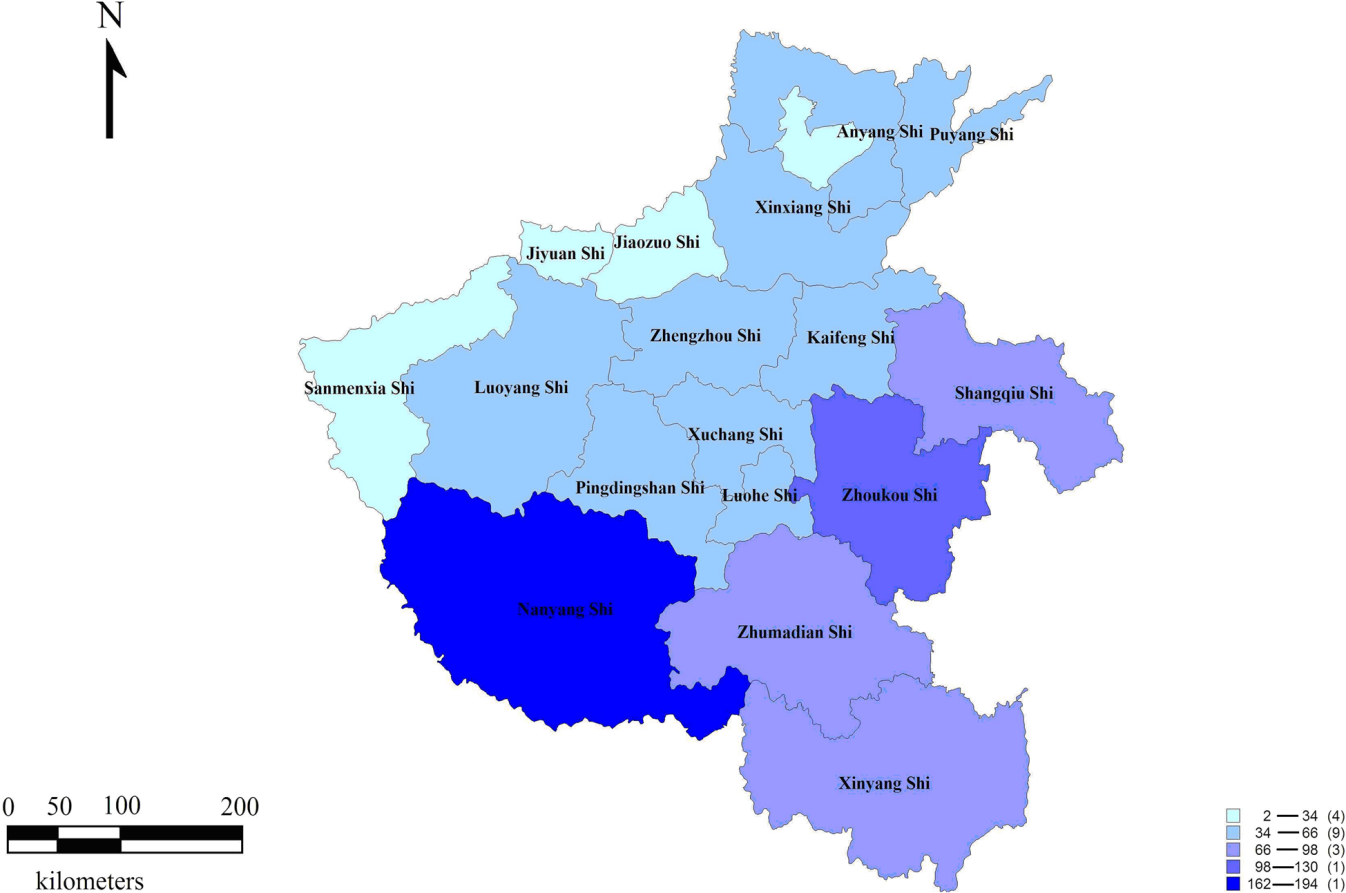
Regional distribution of human rabies in Henan province from 2005 to 2013

Geographically, all municipalities reported patients who had rabies. The majority of cases were distributed in six municipalities (Nanyang, Zhoukou, Shangqiu, Zhumadian, Xinyang and Xuchang) (Fig. 2). These cities, located in the south and east of the province, reported 584 cases accounting for 57.1 % of the total cases. The four cities in the west and north of the province (Sanmenxia, Jiyuan, Jiaozuo and Hebi) only reported 43 cases (4.2 %). Sanmenxia and Jiyuan had reported only six and two cases, respectively, in the nine-year study period. Human rabies in Henan province occurred mainly in the rural areas. The cases in the rural areas comprised 98 % of the cases (*n* = 1001) with an average annual incidence of 0.22/10 million. On the other hand, only 2 % (*n* = 21) of the cases were reported in the city, producing an average incidence of 0.0085/10 million.

### Time distribution of human rabies

**Fig. 3:**
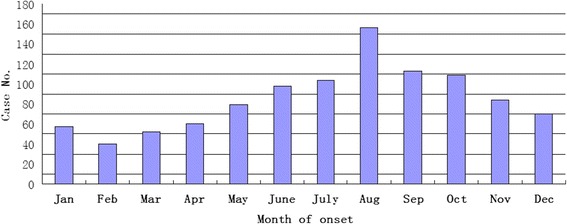
Time distribution of human rabies in Henan province from 2005 to 2013

There were case reports of human rabies in every month. According to the data analysis of the past nine years, the results indicated that the number of cases reached the morbidity peak in August and trough in February (Fig. 3). The disease was most often found in summer (July to September) which accounted for 36.4 % (*n* = 372) of the total number of cases (*χ*^2^ = 145.1, *p* ≤ 0.000) (Table 2).

**Table 2:** Season distribution of reported human rabies cases in Henan province from 2005 to 2013

Season	Total	Percentage (%)
Spring	191	17.6
Summer	360	36.4
Fall	306	30
Winter	165	16
Total	1022	100

### The demographic distribution characteristics of human rabies

Among the rabies patients the male-to-female ratio was 2.41:1 (722 males, 300 females), and age ranged from 1–78 years. Respective totals of 281 cases and 376 cases were reported in the 0–15 and 40–60 age groups, which accounted for nearly 2/3 of the total cases. In relation to patient occupations, farmers, students, unemployed persons, migrants and children comprised, respectively, 76.6, 9.8, 4.8, 4.0 and 4.8 % of the rabies cases (Table 3).

**Table 3:** The demographic characteristics of all the human rabies cases in Henan province (*n* = 1022)

Categories		Total	Percentage (%)
Gender			
	Male	722	70.6
	Female	300	29.4
Age			
	<15	164	16
	~15	67	6.6
	~30	221	21.6
	~45	330	32.3
	~60	207	20.3
	~75	33	3.2
Occupation			
	Farmers	783	76.6
	Unemployed persons	49	4.8
	Migrants	41	4
	Students	100	9.8
	Children	49	4.8

Overall, 430 rabies case questionnaires were collected from 2005–2013 in Henan province. Nearly all the rabies patients (99.1 %) were infected by dog bites. The remaining two patients were infected by being scratched by a cat. Some individuals were infected by slaughtering dogs or by having the anus licked by dogs. Domiciled animals, stray animals, animals from neighbors and unknown source accounted for 46.3 % (199/430), 36.7 % (158/430), 14.4 % (62/430) and 2.6 % (11/430), respectively in all the animals that transmit the disease. Furthermore, 96.2 % of the dogs had received no immunization, versus only 3.8 % who were immunized.

The patient exposure to rabies was classified according to “Standard of Preventive Treatment for Rabies Exposure” published by the Ministry of Health of the People's Republic of China in 2009. Following this, the patient exposure was classified into Category I, Category II, Category III, which accounted, respectively, for 33.5 % (144/430), 38.8 % (167/430) and 22.6 % (97/430), with the remaining cases unknown. After exposure, the wounds were incorrectly processed in 87.7 % (377/430) of the patients. In these patients, the untreated and simple self-treatment were accounted for 67.2 % (289/430) and 20.4 % (88/430). Only 12.3 % (53/430) of patients went to the hospital to accept regular treatment. Also, only 2.6 % (11/430) cases received full course vaccination on time versus 88.3 % (380/430) cases without any vaccination. Category III exposure patients vaccinated against rabies immunoglobulin accounted for only 6.6 % (7/106), the same as other regions [7, 8] (Table 4).

**Table 4:** Comparison of different exposure level processing modes

Exposure Categories	Total	Wound treatment	Vaccine inoculation	Antibody injection
	*n*		Percentage (%)	
Category I	149	36.2	4	0.2
Category II	175	74.2	10.9	3.4
Category III	106	100	23.6	6.6

## Discussion

Henan province is the main epidemic area in China and has been the focus of prevention and control of rabies [9–12]. In the past 55 years, Henan province has posted the peak incidence of human rabies three times [3.4]. Since 2005, the incidence of rabies presented a rapid rising trend and remained high during 2007–2010, showing an epidemic periodic on the long-term trends. A previous survey indicated that the rabies epidemic areas of Henan province were in the south and east portion of the province, such as Xinyang, Zhumadian, Nanyang, Shangqiu and Zhoukou. The recent concern is the epidemic area in the county level overall was increasing in Henan province during 2005–2013. This rise may be associated with the dog population dynamics. This tendency is now spreading rapidly, which indicated that the source of the infection has been spreading.

The fact that 76.6 % of farmers had rabies was in accord with the fact that most patients lived in rural areas. This characteristic of the patients in the current study was similar to the rabies epidemic characteristics in other areas [13–17]. In the vast rural areas, young adults go out to work while leaving the elderly and children at home. It is more likely that these families raise the dog for guarding the house. These animals are mostly backyard dogs and not vaccinated. Children who were bitten may not tell their grandparents and thus received no treatment. This may be related to the increased number of children reported with rabies in recent years. Studies have shown that pre-exposure prophylaxis of rabies vaccine can effectively protect the children bitten by animals, and significantly reduce the risk the incidence of rabies in children, and serve as an effective measure for rabies elimination work [18].

Research shows that these measures can effectively reduce the rabies incidence in a manner similar to timely disposal of wound dressings, vaccination and passive immunity preparation injection after exposure. In our study, nearly 88.3 % patients had given up rabies vaccination after exposure and only 6.6 % (Category III patients) used rabies immunoglobulin. This is likely cause of the high incidence of rabies in China. At present, the whole course vaccination against rabies expenses costs approximately U$ 25-85/person, while passive immunization costs U$ 35/person (anti-rabies serum) to U$ 170/person (anti-rabies immune globulin). Some persons exposed to rabies, especially in rural areas, were not vaccinated because they could not afford the expensive vaccine [10]. If the government would include the rabies vaccine and passive immune agents on the list of medicines covered by medical insurance, it may effectively reduce the economic burden of residents, promote the post exposure prophylaxis (PEP) and effectively reduce the incidence of rabies.

In 2006, the estimated number of dogs in China was over 75,000,000. According to the statistical data the vaccination rate of dogs in some areas was lower than 15 % in other provinces [18]. As the vaccination rate of dogs was only 3.8 %, Henan province is facing the risk of a more severe rabies epidemic.

The Pan American Health Organization (PAHO) has achieved the goal of eradicating rabies by the development of a mass canine rabies vaccine in Latin American countries [19–21]. These successful experiences suggest that the key to rabies prevention and control is the virus control. Based on the “one health” concept [22], first we should conduct a baseline survey and investigate the risk factors for rabies incidence, then construct the “one health” mode in the local community. On the one hand, health education and pre-exposure prophylaxis of rabies vaccine in children must be conducted; on the other hand, a mass vaccination program should be conducted in local canines. After several years of construction, it can effectively reduce the incidence of rabies in local residents. But there are two problems in Henan that hamper the conducting of this program; first no one knows the dog situation of residents in Henan province, including the number of dogs, dog density, residents that promote immunization for their animals; secondly, there is no veterinary rabies vaccine production in China, while the vaccine is completely dependent on importation and its price is high. Furthermore, it is logistically difficult to promote it in the rural areas. These two aspects are crucial to our future research direction. If the two above mentioned problems were to be solved, we could effectively promote the “one health” community mode and eliminate rabies.

## Conclusion

In China, cases of rabies increased in Henan province during the period from 2005–2013. The standardized disposal of wound dressings, vaccination and passive immunotherapy after exposure are important factors to avoid mortality of patients. Furthermore, a national animal vaccination program is crucial. Finally, the disease could be better managed by an integrated surveillance policy between human health and veterinary sectors in association with improved education and better government policies.
